# Full-Scale Measurements and System Identification on Sutong Cable-Stayed Bridge during Typhoon Fung-Wong

**DOI:** 10.1155/2014/936832

**Published:** 2014-06-03

**Authors:** Hao Wang, Tianyou Tao, Tong Guo, Jian Li, Aiqun Li

**Affiliations:** ^1^Key Laboratory of C&PC Structures of Ministry of Education, Southeast University, No. 2 Sipailou, Nanjing 210096, China; ^2^School of Civil Engineering, Southeast University, No. 2 Sipailou, Nanjing 210096, China; ^3^Department of Civil, Environmental & Architectural Engineering, The University of Kansas, 1530 West 15th Street, Lawrence, KS 66045, USA

## Abstract

The structural health monitoring system (SHMS) provides an effective tool to conduct full-scale measurements on existing bridges for essential research on bridge wind engineering. In July 2008, Typhoon Fung-Wong lashed China and hit Sutong cable-stayed bridge (SCB) in China. During typhoon period, full-scale measurements were conducted to record the wind data and the structural vibration responses were collected by the SHMS installed on SCB. Based on the statistical method and the spectral analysis technique, the measured data are analyzed to obtain the typical parameters and characteristics. Furthermore, this paper analyzed the measured structural vibration responses and indicated the vibration characteristics of the stay cable and the deck, the relationship between structural vibrations and wind speed, the comparison of upstream and downstream cable vibrations, the effectiveness of cable dampers, and so forth. Considering the significance of damping ratio in vibration mitigation, the modal damping ratios of the SCB are identified based on the Hilbert-Huang transform (HHT) combined with the random decrement technique (RDT). The analysis results can be used to validate the current dynamic characteristic analysis methods, buffeting calculation methods, and wind tunnel test results of the long-span cable-stayed bridges.

## 1. Introduction

Sutong cable-stayed bridge (SCB), the longest cable-stayed bridge in the world when it is open to traffic in 2008, is regarded as the engineering achievement which makes the main span of cable-stayed bridges develop from a few hundred meters to thousand meters in the last few decades. The structural stiffness of a long-span cable-supported bridge drops significantly with the increase in the span length, which makes the wind-induced vibration particularly important to bridge safety. In 1940, the Old Tacoma suspension bridge accident in the United States caused by the wind-induced flutter made the government and research institutes fully aware of wind-induced disaster for cable-supported bridges. However, the trend toward increasing bridge span length and deck width did make the wind-induced buffeting effect more and more prominent to cable-stayed structures, so the field monitoring on wind environment conditions and wind-induced buffeting response becomes an important topic in wind engineering research [1–6]. It is of great significance to conduct full-scale measurement on existing bridges, which is an important and practical method for wind engineering research, capable of validating the reliability of existing buffeting theories, determining key parameters of current buffeting response calculation techniques and exploring the bridge buffeting behavior and mechanism [7–12].

The structural health monitoring system (SHMS) integrating with anemometers and vibration sensors is installed on lots of long-span bridges all over the world at the present time. SHMS also provides a good platform for case studies of the research on buffeting responses of full-scale long-span bridge structures [13]. However, further research and analysis on measured data processing methods are required in order to carry out the refinement analysis on bridge buffeting responses and validate the credibility of current buffeting response control methods and techniques. As Typhoon Fung-Wong passed through China from 29 to 30 July 2008, the full-scale measurements on strong wind characteristics were carried out by using the high-precision three-dimensional ultrasound anemometers, and the typhoon samples through the bridge spot were obtained. Based on the data recorded by anemometers and accelerometers in the SHMS of SCB, the measured buffeting response characteristics of some key components under Typhoon Fung-Wong were investigated in the paper. The results can provide the measured information for wind-induced buffeting safety assessment of SCB based on the SHMS and the reference values for wind-resistant design of other similar long-span cable-stayed bridges.

## 2. Project Background

**Figure 1:**
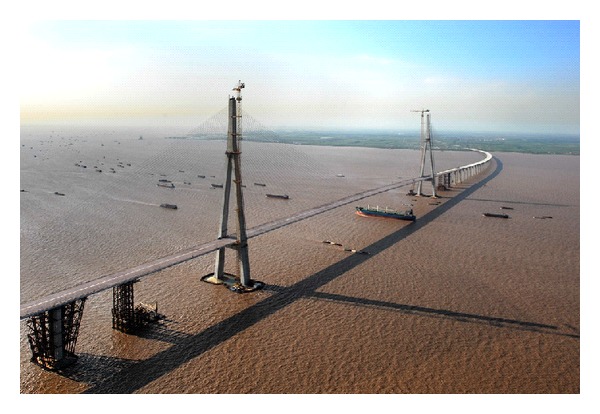
View of Sutong cable-stayed bridge (SCB).

The studied structure in this paper, SCB, is located in the eastern part of Jiangsu province between Nantong and Suzhou, as shown in Figure 1. According to the highest construction standards, SCB is a super-long-span cable-stayed bridge constructed by the complicated and advanced modern techniques. Now, SCB is the second longest cable-stayed bridge in the world and a record-breaking project in the bridge-building history.

**Figure 2:**
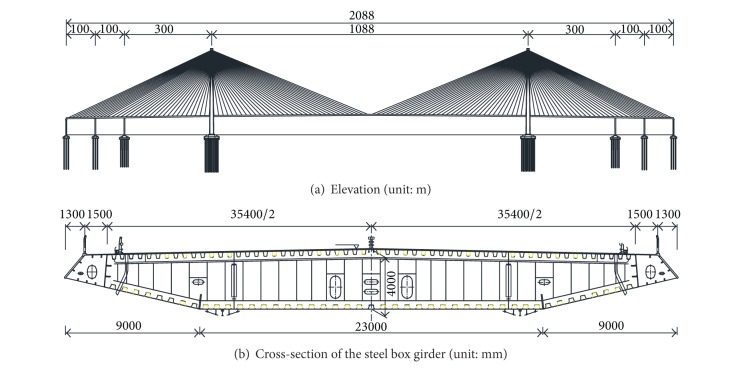
Configurations of Sutong bridge.

SCB is a double-tower double-cable-plane steel-box-girder cable-stayed bridge with a main span of 1088 m, as shown in Figure 2(a). Streamline flat steel-box-girder is employed as bridge deck, as shown in Figure 2(b). The total width of the girder is 41.0 m. Without wind mouth, the width of the roof is 35.4 m, the bottom is 9.0 + 23.0 + 9.0 m, and the height of the deck at centerline is 4.0 m. Parallel wire cables are adopted for structural suspension system, and the typical distance between each cable on the deck is 16 m. The back cable is in the side span, and the distance between cables on towers is 2 m. The number of cables on the bridge totally is 4 × 34 × 2 = 272. The longest length of the cable is about 577 m, and the largest size of the cable is PES7-313. Main towers are inverted Y-shaped structures, including upper tower columns, middle tower columns, lower tower columns, and lower beams. The height of main towers is 300.4 m, which is 230.41 m height above the bridge deck. Between main towers and the deck, there are horizontal wind-resistant bearings and vertical viscous dampers. The type of main tower foundation is 131 bored piles with the inner diameter of 2.8 m. The layout of piles is quincunx and 117 m in length. The pile caps are dumbbell-shaped; the plane size of each pile cap is 51.35 m × 48.1 m; and the thickness is shifted from 5 m on the edge to 13.324 m. The size of connection beam between two pile caps is 11.05 m × 28.1 m and 6 m in thickness.

## 3. Description of Typhoon Fung-Wong

SCB is located in the downstream near the estuary of the Yangtze River. The climate zone of the bridge site is in midlatitude region and is part of subtropical southern moist monsoon climate. The monsoon circulation mainly dominates the climate of this area, which is mild, for four distinct seasons with abundant rainfall; as a result, the climate is neither like that of inland areas nor that of marine areas. The typhoon during summer season likely induces the major critical source of wind loads for bridge structures.

**Figure 3:**
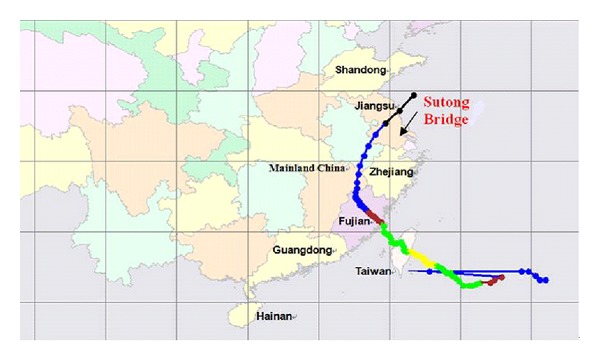
Moving track of Typhoon Fung-Wong.

**Figure 4:**
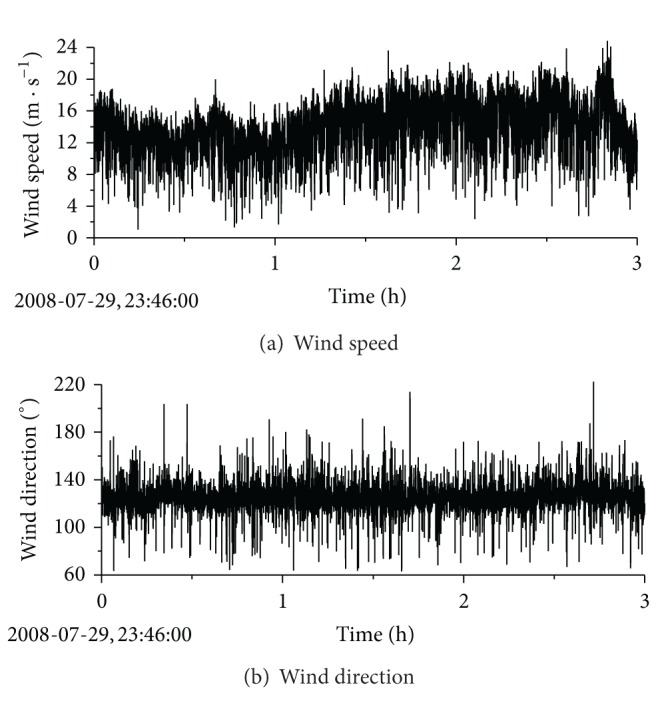
Samples of Typhoon Fung-Wong.

Reported by Taiwan Central Weather Bureau, the Typhoon Fung-Wong developed and was defined as a tropical depression on 25 July 2008 with its intensity increased continuously. Typhoon Fung-Wong entered into Taiwan at 6:50 local time (UTC+8) on 28 July and arrived Fujian province at 23:10 on 28 July. On the afternoon of 29 July, Typhoon Fung-Wong attacked the southeastern part of the Jiangsu province, and it finally weakened into tropical storm around 15:00 on 31 July. Measured wind data showed that it went across the site of Sutong bridge between 23:46 on 29 July and 2:46 on 30 July.  Figure 3 shows the route of Typhoon Fung-Wong. Fortunately, the strength of Typhoon Fung-Wong was moderate and it did not hit the Sutong bridge directly.  Figure 4 shows the typical samples of the simultaneous measurements of Typhoon Fung-Wong from SHMS at the midspan of SCB.

## 4. Full-Scale Measurements on Sutong Bridge during Typhoon Fung-Wong

### 4.1. Structural Health Monitoring System of SCB

**Figure 5:**
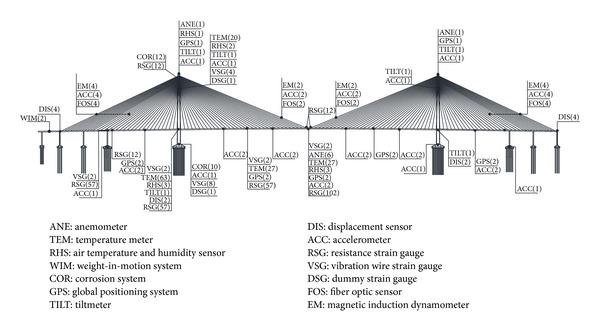
Layout of sensors in SHMS of SCB.

Taking into account the importance and research value of SCB structure, SHMS is employed to predict and assess the health condition of SCB during both construction stage and operation stage. The overall sensor layout is shown in Figure 5.

Four 3D anemometers are installed in the SHMS of SCB. Two anemometers are employed separately on the top of the south and north tower each; the other two are placed on the upstream and downstream side of the middle of the deck. These anemometers on the SHMS can provide the measured data of wind environment at the bridge spot. Forty dual-axis accelerometers and six triple-axis accelerometers were integrated in the SHMS of SCB to monitor vibration responses of key components, such as the deck, cables, and main tower structures. This paper focuses on the analysis of the data recorded by the accelerometers installed on the deck and cables under the typhoon condition.

### 4.2. Additional Field Measurements on Typhoon Fung-Wong

**Figure 6:**
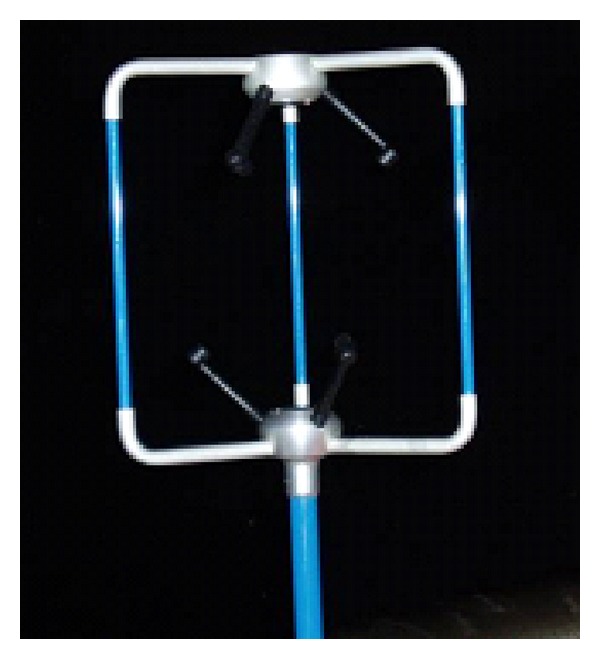
3D sonic anemometer.

The SHMS provides only average or maximum wind speed and direction data. It does not collect continuous time history wind speed and direction data when the frequency is greater than 1 Hz. Because of this limitation, the 1590-PK-020 three-dimensional ultrasonic anemometer with high sampling frequency and precision was installed in the midspan of SCB for the additional field measurements during strong winds. The 1590-PK-020 anemometers are produced by the Gill Instruments Limited, as shown in Figure 6.

**Figure 7:**
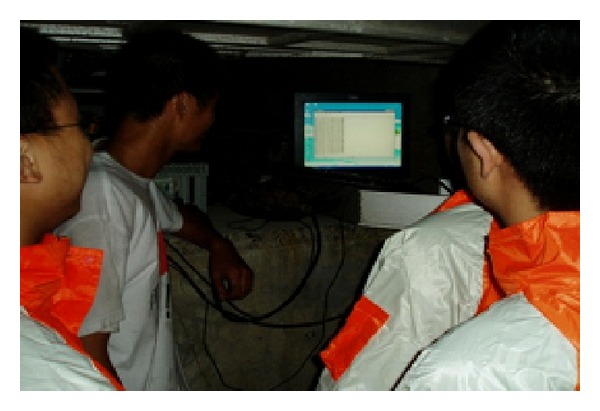
Adjusting the measurement instruments.

Figure 7 shows the photo of adjusting the measurement instruments during Typhoon Fung-Wong. The operational temperature of anemometers was −40∼+70°C, available sound velocity was 300∼370 m/s, and the allowable humidity for the instrument was 5%∼100%. In order to avoid the excess of measured data caused by the too-long recording time, the sampling frequency in this study was set to 20 Hz. For accurately measuring the wind direction variation in typhoon period, polar coordinate system was selected as the output mode of data. The wind speed measuring range for the instrument in the test was set to 0∼45 m/s; the measurement accuracy was set to 0.01 m/s; the wind direction range was set to 0∼359.9° with 0.1° as the measurement accuracy. North wind was defined as 0° for anemometers data, and clockwise rotation is defined as positive direction. For the buffeting response analysis of SCB, the north direction of anemometers was set to point at Nantong along the axis of the bridge when installed. Since there was a 10.6° angle difference between the North-South direction and the longitudinal axis of the bridge, the actual wind direction is the sum of the measured wind direction and the angle difference.

## 5. Measurement and Analysis of Typhoon Fung-Wong at Bridge Site

Compared to the anemometers installed in the SHMS, the anemometers used in the field measurements have higher frequency and accuracy. Therefore, the wind data recorded at the bridge spot by using the 1590-PK-020 anemometer during Typhoon Fung-Wong were selected for analysis in this study. Consequently, the comparison and the verification of wind characteristics between SHMS results and additional field measurements were conducted, which indicates that the maximum values, the mean values, and the turbulence wind parameters are in good agreement during the same period.

### 5.1. Wind Speed and Direction

**Figure 8:**
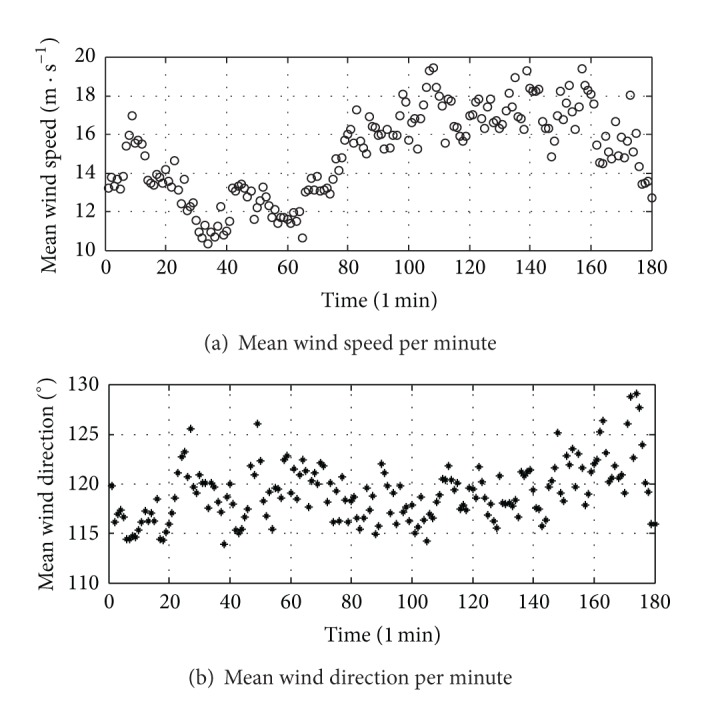
Mean wind speed and the direction of Typhoon Fung-Wong.

Measured data indicates that wind speeds during 23:46 on 29th July to 2:46 on 30th July are relatively stable and large. As a result, the characteristics of strong wind during the three hours are selected for analysis. The time interval is 1 min in the analysis and there are total 180 time intervals. The mean wind speed and direction of Typhoon Fung-Wong when it passed through the SCB spot are shown in Figures 8(a) and 8(b), respectively.

Figure 8 shows that the mean wind speeds per minute during Typhoon Fung-Wong at the bridge spot in the selected time period are in the range of 10 m/s to 20 m/s, the mean wind speed in the selected period is 15.02 m/s, and the maximum value is 19.48 m/s at the point 108 min corresponding to 01:34 on 30th July. The main wind direction is ESE and it mainly changes from 115° to 124°. Compared with the other typhoons [11], the wind speed variation during Typhoon Fung-Wong is relatively small, and the wind direction is stable. It is clear that the wind speed reaches the peak value for around 70 minutes from the measured data at point 90 min to those at point 160 min (corresponding time is from 1:16 to 2:26 on 30th July) in the selected time period, and the variation of the wind speed and direction is relatively small. Therefore, this time period can be chosen as the case study for full-scale measurement on buffeting response of SCB during Typhoon Fung-Wong.

### 5.2. Turbulence Intensity

As one of the key parameters determining the turbulence wind load acting on the structure, turbulence intensity reflects the intensity of the fluctuating wind, which can be calculated by

(1) $$\begin{array}{c} I_{i}=\frac{\sigma_{i}}{U}\;\left(i=u,v\right), \end{array}$$

where *U* is the horizontal average wind speed; *σ*_*i*_ is the standard deviation of the fluctuating components during the user-defined averaging time interval.

**Figure 9:**
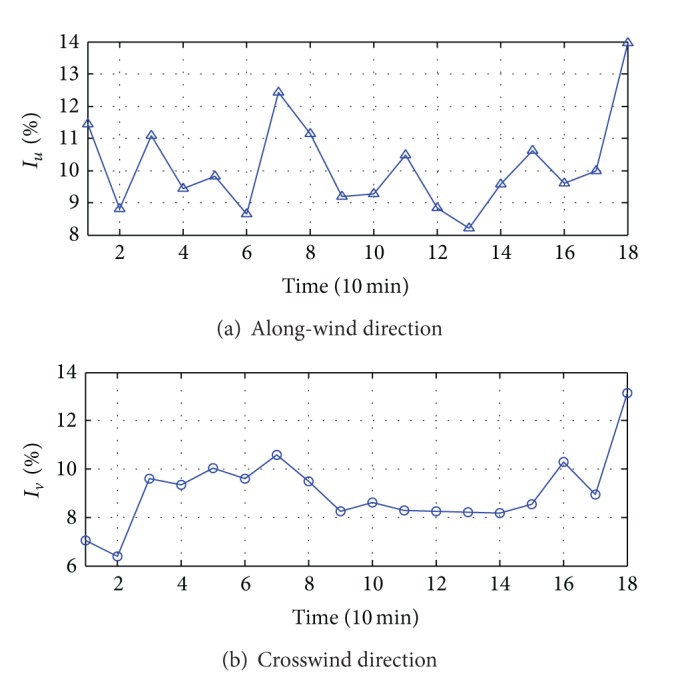
10 min turbulence intensity of Typhoon Fung-Wong.

During the Typhoon Fung-Wong, the along-wind and across-wind turbulence intensity (*I*_*u*_ and *I*_*v*_) were calculated with time interval of 10 minutes, and the results were shown in Figure 9.

**Figure 10:**
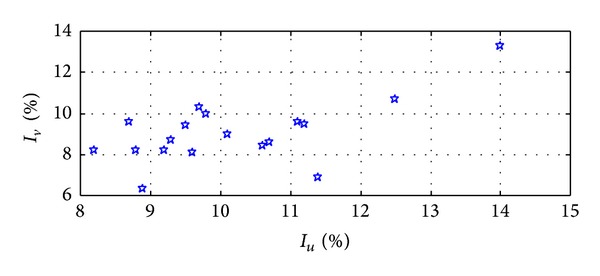
Relationship between *I*_*u*_ and *I*_*v*_ of Typhoon Fung-Wong.

As shown in Figure 9, the mean values of *I*_*u*_ and *I*_*v*_ of Typhoon Fung-Wong at bridge site were 10.14% and 9.04%, respectively, whereas the maximum values of *I*_*u*_ and *I*_*v*_ were 13.96% and 13.13%, respectively. The current wind-resistant design specification for China highway bridges [14] suggests that the *I*_*u*_ at 50∼70 m above ground at the open-sea field should be about 11%. Obviously, the measured results were a little lower than the specification value. Meanwhile, the specification suggests that *I*_*v*_ should be equal to 0.88 *I*_*u*_ when there are no measured data. In this study, *I*_*v*_ was equal to 0.89 *I*_*u*_ based on the mean values of the measured turbulence intensity, which showed that the measured results are well consistent with this rule. Figure 9 also indicates that there was an obvious correlation between the *I*_*u*_ and the *I*_*v*_, and both of the turbulence intensity maximums appeared at the 18th data point, which is shown in detail in Figure 10.

### 5.3. Turbulence Integral Length Scale

**Figure 11:**
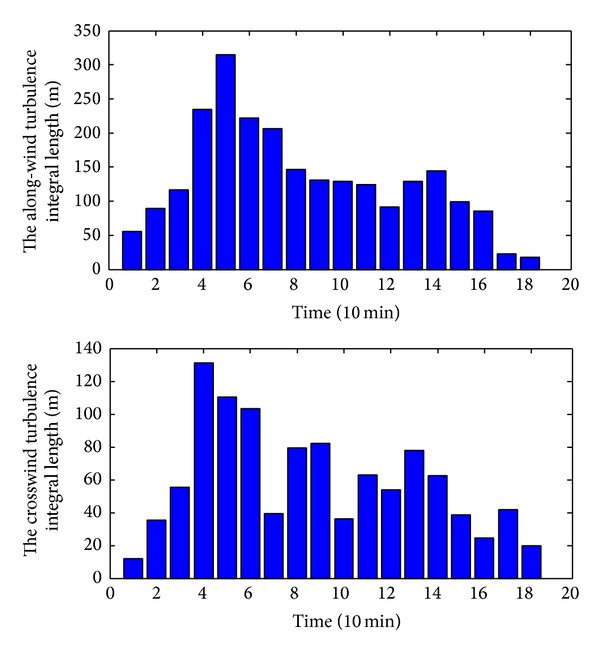
Turbulence integral length scale of Typhoon Fung-Wong.

The turbulence integral length scale of the boundary layer of the atmosphere usually fluctuates in a wide range. Based on the measured wind speed and direction with time interval of 10 minutes, the turbulence integral length scale was calculated using the autocorrelation function integral method as follows [1]:

(2) $$\begin{array}{c} L_{i}^{x}=\frac{U}{\sigma_{i}^{2}}\overset{\infty }{\underset{0}{\int }}R_{i}\left(\tau \right)d\tau \;\left(i=u,v\right), \end{array}$$

where *U* is the horizontal average wind speed; *σ*_*i*_ is the standard deviation of velocity fluctuations; and *R*_*i*_(*τ*) is the autocorrelation function of turbulence component *i*; the upper limit of integration uses the value of *τ* when the corresponding correlation coefficient decreases to 0.05. The along-wind and crosswind turbulence integral length scale, *L*_*u*_^*x*^ and *L*_*v*_^*x*^, were shown in Figure 11, respectively.

As shown in Figure 11, most of the *L*_*u*_^*x*^ values range from 80 m to 220 m. The maximum, mean, and minimum *L*_*u*_^*x*^ values were 314.7 m, 131.5 m, and 18.8 m, respectively. The *L*_*v*_^*x*^ value changes mainly from 30 m to 100 m. The maximum, mean, and minimum *L*_*v*_^*x*^ values were 131.0 m, 59.3 m, and 11.9 m, respectively. The comparison results among the Typhoons “Fung-Wong,” “Matsa,” and “Wipha” [4] show that the turbulence integral length scales of different strong winds differ greatly. The turbulence integral length scales for each typhoon are case-sensitive.

### 5.4. Turbulence Power Spectrum Density

The specification in China [14] adopts the horizontal wind spectrum expression suggested by Kaimal in 1972 as the design spectrum. The wind speed at *Z* altitude is *U*; then the along-wind turbulent wind power spectrum density function is defined as

(3) $$\begin{array}{c} \frac{nS_{u}\left(n\right)}{{\left(u^{*}\right)}^{2}}=\frac{200f}{{\left(1+50f\right)}^{5/3}}. \end{array}$$

In (3), *S*_*u*_(*n*) is the along-wind spectral density function, *n* is the natural fluctuation frequency,  *f* is the Monin coordinate and *f* = *nZ*/*U*, and u* is the airflow friction speed. Because there are no measured data of u*, the variance of corresponding fluctuating component of wind velocity can be calculated by the energy unitary method as [15]

(4) $$\begin{array}{c} \sigma_{u}^{2}=6{\left(u^{*}\right)}^{2}. \end{array}$$

**Figure 12:**
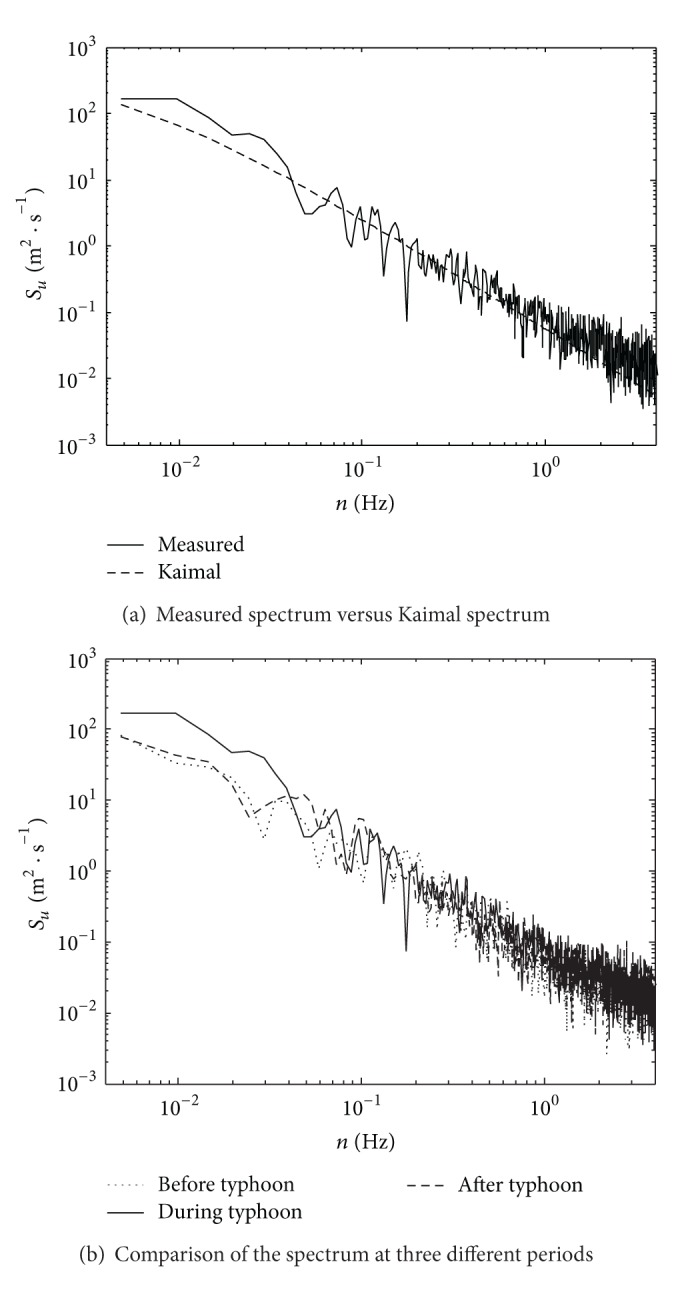
Analysis of along-wind turbulence power spectrum of Typhoon Fung-Wong.

The comparison of the measured along-wind turbulence power spectral density function at the bridge site and the Kaimal spectrum is shown in Figure 12(a), where the value of (u*)^2^ is 0.968 (m/s)^2^ obtained by (4), and spectral analysis is then calculated by using the maximum 10 min wind speed. The Hamming window was adopted to reduce the signal leakage in the frequency domain caused by signal truncation in the time domain, and the segment smoothing technique was used to reduce the random error of spectral estimates. The comparison of the along-wind turbulence power spectrum at the period before typhoon (the mean wind speed is 12.28 m/s), the period during typhoon (the mean wind speed is 15.02 m/s), and the period after typhoon (the mean wind speed is 13.98 m/s) is shown in Figure 12(b).

As shown in Figure 12(a), the measured along-wind turbulence power spectrum density of Typhoon Fung-Wong coincided well with the Kaimal spectrum in general. Compared with Kaimal spectrum, the measured spectrum coincided well with it in the middle frequency region from 0.04 Hz to 0.25 Hz, and it was a little higher in both the low frequency and the high frequency regions, which is the difference between the distribution characteristic of the measured wind spectrums of the Typhoons “Matsa,” “Khanun,” and “Wipha” [4]. Figure 12(b) shows that, with the increase of the mean wind speed during typhoon, the measured turbulence power spectrum value became larger, which was consistent with the existing conclusions.

Since the measured spectrum curve of Typhoon Fung-Wong was a little higher in both the low and the high frequency regions, therefore, the curve expression with inflection points should be employed when fitting the curve. If the expression in (2) to fit the curve was selected, the inflection points that exist in the along-wind turbulence power spectrum of Typhoon Fung-Wong cannot be reflected. Since the turbulence power spectrum density function is the basis of wind field simulation, therefore, it is essential to propose a new spectrum expression for accurately simulating the three-dimensional turbulence wind field at bridge site.

The vertical turbulence power spectrum function often adopts the Panofsky spectrum:

(5) $$\begin{array}{c} \frac{nS_{w}\left(n\right)}{{\left(u^{*}\right)}^{2}}=\frac{6f}{{\left(1+4f\right)}^{2}}. \end{array}$$

**Figure 13:**
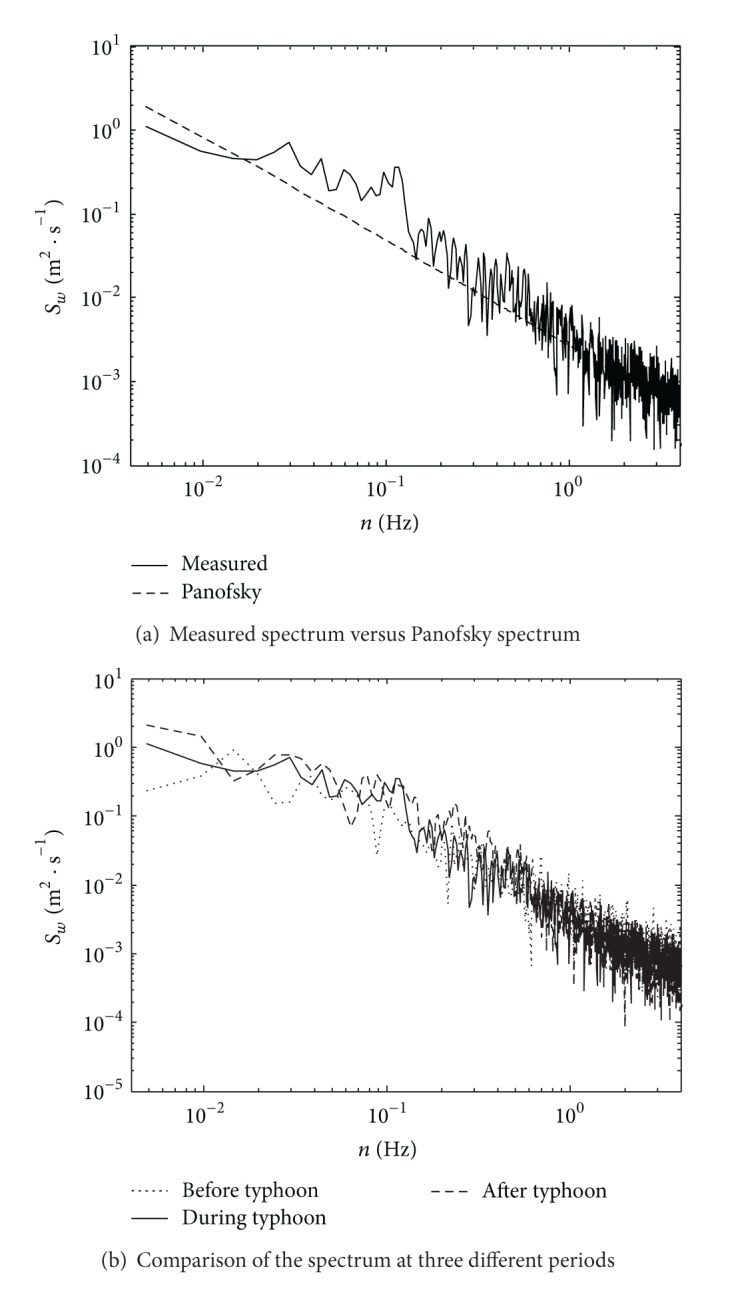
Analysis of the vertical turbulence power spectrum of Typhoon Fung-Wong.

In (5), *S*_*w*_(*n*) is the vertical spectral density function; the other symbols are identical to those defined in (2). The comparison between the measured vertical turbulence power spectrum function and Panofsky spectrum can be seen in Figure 13(a), and the comparison of the vertical turbulence power spectrum at the period before typhoon, during typhoon, and after typhoon is shown in Figure 13(b).

As indicated in Figure 13(a), the measured vertical turbulence power spectrum density of Typhoon Fung-Wong did not fit well with the Panofsky spectrum. The measured spectrum was higher than the Panofsky spectrum in the middle frequency domain from 0.02 Hz to 1 Hz, which was similar to the measured results from the SHMS. Therefore, the discrepancy of Typhoon Fung-Wong was mainly caused by the characteristic of the Typhoon Fung-Wong itself. Figure 13(a) also shows that the measured vertical spectrum expression still has inflection points while the Panofsky spectrum doesn't have. If (4) was used to fit the measured spectrum curve by revising the correlation parameters, however, it is difficult to make the measured spectrum coincide perfectly with the Panofsky spectrum in anyway. In addition, Figure 13(b) also shows that, due to the characteristic of Typhoon Fung-Wong itself, the measured vertical turbulence power spectrum values were not obviously influenced by wind speed, which is quite different from other typhoons [11]. The above wind characteristics of Typhoon Fung-Wong should be paid attention to during the wind-induced buffeting analysis.

## 6. Buffeting Analysis of SCB Based on Field Measured Data

The 8310-type acceleration sensors, produced by the Kistler Company in Switzerland, are used in the SHMS of SCB. Referring to the sensor parameters, the allowable operation temperature ranges from −40 to +85°C and the setup sampling frequency is 20 Hz. Each sensor was carefully calibrated before installation. During Typhoon Fung-Wong, acceleration responses at the key sections were real-time recorded. Since the influence of temperature and vehicle load was relatively little at night, the data from 1:16 to 2:16 on 30th July 2008 was analyzed.

### 6.1. Buffeting Acceleration Response Analysis of the Deck Based on Measured Data

**Figure 14:**
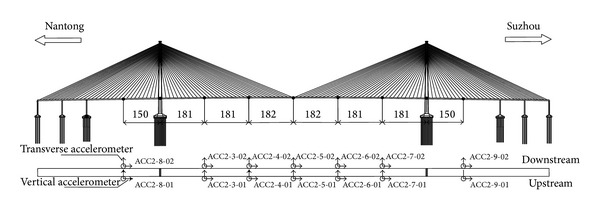
Layout of sensors for deck vibration monitoring in SCB.

In the SHMS of SCB, the vibration responses at seven key sections of the deck were selected to be monitored, including five sections at the main span and two sections at the side span. The layout of sensors in each section is shown in Figure 14.

**Figure 15:**
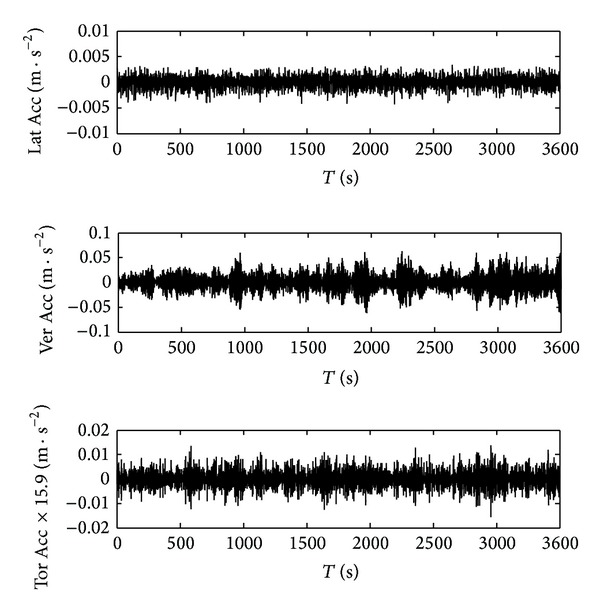
Measured lateral, vertical, and torsional accelerations.

In this paper, the measured acceleration responses at the midspan section of the deck (section ACC2-5) were analyzed, as shown in Figure 15. The lateral (Lat) and vertical acceleration (Ver Acc) responses were the average of recorded data from two sides of the same section. Torsional acceleration (Tor Acc) response was obtained from dividing the difference of two measured vertical acceleration data by 31.8 m (distance between the two sensors). For convenience of comparison, the torsional acceleration responses were multiplied by 15.9 m (half of the distance between the two vertical sensors).

#### 6.1.1. The Measured Acceleration Response RMS Value of the Deck

**Figure 16:**
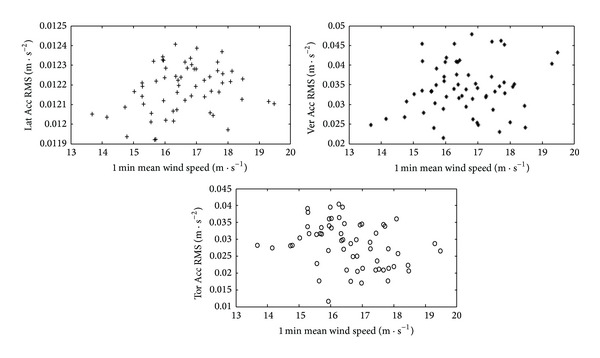
Variation of deck acceleration response RMS at ACC2-5 versus mean wind speed.

In order to analyze the relationship between the measured acceleration response and the wind speed, the 1 min measured acceleration response RMS values of section ACC2-5 was calculated. The relationship between the 1 min RMS values of measured lateral, vertical, and torsional acceleration responses of the deck and the 1 min mean wind speed is plotted in Figure 16. As shown in Figure 16, the lateral, vertical, and torsional acceleration response RMS values of the deck generally increase with wind speed. However, some randomness appears because of the influence of the stochastic factors such as the wind direction and the spatial correlation. Without the decomposition of wind direction, it is difficult to obtain an effective evaluation equation based on the relationship of the measured acceleration RMS values and the wind speed.

#### 6.1.2. Spectral Analysis of Measured Acceleration Response of the Deck

**Figure 17:**
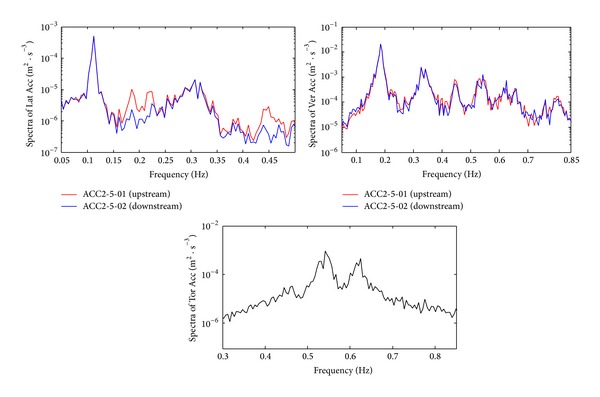
Spectra of deck acceleration response at the middle of the deck.

Spectral analysis on lateral, vertical (upstream and downstream), and torsional acceleration responses at the midspan of the deck was carried out by means of FFT technique and the analysis results are shown in Figure 17. In the analysis, the Hamming window was used to reduce the signal leakage in the frequency domain and the piecewise smoothing technique was used to reduce the random error of spectral estimates. 1-hour sample data (72000 numbers) were divided into 19 subsegments. The length of subsegment was selected as 6 min (7200 numbers) and the overlapped length was 3 min (3600 numbers). Figure 17 shows that the measured results of upstream and downstream sides at the same section were in agreement, so the credibility of sensors was validated.

The natural frequencies of the Sutong bridge are obtained from the spectral analysis [16]. The natural frequencies obtained by the FE method, the measured frequencies during Typhoon Fung-Wong, and normal windy conditions are compared. The 1st lateral bending, vertical bending, and torsional vibration of the deck were listed in Table 1. Table 1 also shows the modal damping ratios identified during different wind conditions.

As shown in Table 1, all of natural frequencies of the first mode during Typhoon Fung-Wong are lower than those during normal windy conditions, which is consistent with the phenomenon presented by Siringoringo and Fujino [17]. The *E*_*f*2_ values exhibit that the measured first vertical, lateral bending, and torsional modes of the deck during Typhoon Fung-Wong could match the FE calculated modes well, and all four *E*_*f*2_ values are less than 5%. Therefore, the existing FE model is capable of carrying out structural analysis reliably. In addition, the *E*_*f*2_ value associated with the lateral bending mode is relatively large, which is mainly due to the horizontal wind-resistant bearings installed on SCB. These bearings are used to limit the transverse displacement of the steel-box girder. However, the constraint of bearings could not be accurately simulated in numerical calculation. It can be proved that the bearings play an important role in dynamic characteristics of long-span cable-stayed bridges.

The Hilbert-Huang transform (HHT) combined with the random decrement technique (RDT) is used to identify the modal damping ratios of the SCB based on the measured deck acceleration response. Firstly, the measured acceleration response is decomposed into a series of intrinsic mode functions (IMF) using the empirical mode decomposition (EMD) method. The free vibration response of associated modes of the SCB is then obtained using the RDT. Finally, the total damping ratios of the associated vibration modes are identified from the free vibration response by the Hilbert transform [18]. Based on the aerodynamic damping estimated from the wind tunnel tests [1] at Tongji University, the modal damping ratio of SCB is finally obtained by deducting the corresponding aerodynamic damping ratio from the identified total damping ratio.

Table 1 also shows that all of the natural damping ratios of the first mode during Typhoon Fung-Wong are higher than those during normal wind, which is also consistent with the research results by Siringoringo and Fujino [17]. In addition, the identified *ζ* values corresponding to the torsional vibration modes are relatively large, which should be paid special attention to when modeling the long-span cable-stayed bridges.

### 6.2. Buffeting Response Analysis of the Cables Based on Measured Data

#### 6.2.1. The RMS Value of Measured Cable Acceleration Response

**Figure 18:**
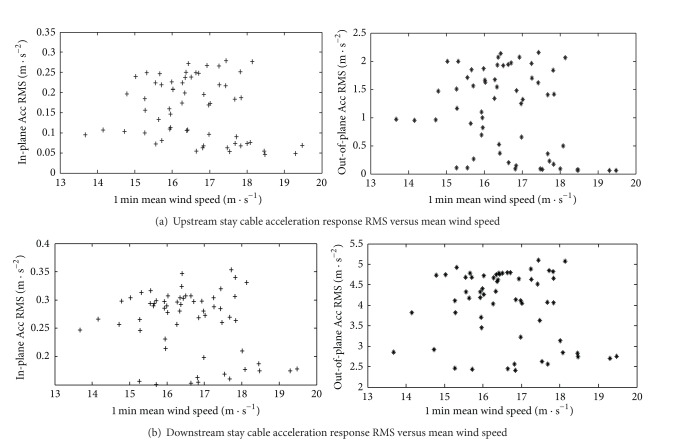
Acceleration response RMS of stay cable versus mean wind speed.

Since the wind-rain induced vibration mechanism of cable still needs to be clarified and most of the existing researches focus on the wind tunnel tests [19–23], the field tests on the cable vibration responses are of great significance. 12 cables are selected for monitoring in the SHMS of SCB, as shown in Figure 5. Similar to the analysis of the deck, the measured data of in-plane and out-of-plane acceleration responses of cables were analyzed at the same time. The measured acceleration responses of the longest cable of SCB (section ACC2-11) were analyzed here. In order to compare with each other conveniently, the measured results of upstream and downstream were not averaged. The relationship between the 1 min acceleration response RMS values of section ACC2-11 and the 1 min mean wind speed is shown in Figure 18.

As can be seen in Figure 18, the in-plane and out-of-plane acceleration response RMS values of upstream and downstream stay cables increased with the wind speed. Moreover, some randomness appears which is in agreement with the existing research results. For the same section, the in-plane and out-of-plane vibration characteristics of both the upstream and the downstream cables exhibit a strong correlation. Figure 18 also indicates that, compared with the existing results [24], both the in-plane and the out-of-plane vibration responses of cables of SCB under Typhoon Fung-Wong are small. This is owing to the control effects of the dampers installed near the anchor ends of cables. In addition, Figure 18 shows that the control effects of dampers on the in-plane vibration are obviously better than those on the out-of-plane vibrations.

#### 6.2.2. Spectral Analysis of Cable Measured Acceleration Response

**Figure 19:**
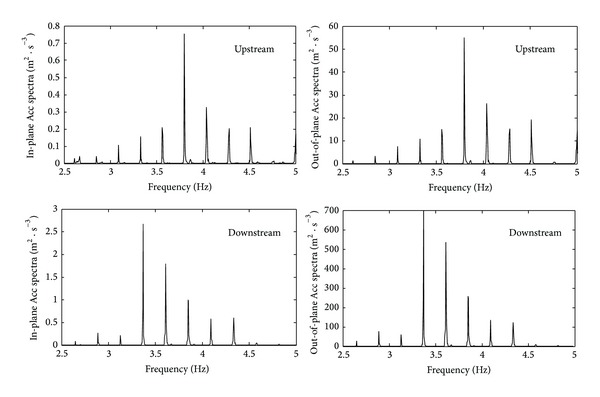
Spectra of stay cable acceleration response.

Similar to the analysis of the deck, spectral analysis on in-plane and out-of-plane acceleration responses was carried out by using FFT methods. Linear coordinates were employed as the transverse and longitudinal axes for spectral analysis on cables, while the combination of logarithmic coordinates and linear coordinates was employed for the spectral analysis on the data of deck. Therefore, it is easy to identify the cable vibration frequencies. The spectral analysis results of the upstream and downstream cables were compared, as shown in Figure 19.

As shown in Figure 19, there was a clear discrepancy between the in-plane and out-of-plane acceleration response amplitudes on the same cable. However, the in-plane and out-of-plane vibration characteristics were very close to each other; the frequency values of each order were almost the same, and the contribution of each order frequency component to vibration responses of cables was also close to each other. Although upstream frequency values are also close to the values of downstream cable, the contribution of each order frequency component to vibration responses of cables was different. The frequencies above 3.75 Hz played a significant role in the contribution of upstream cable vibration responses, while the frequencies above 3.38 Hz dominated the vibration responses of the downstream stay cables. Considering the structural symmetry of SCB, it can be concluded that the wind environment of the windward and leeward cables was different because of the wake effects.

## 7. Conclusions

1. The specification suggests that *I*_*v*_ should be equal to 0.88*I*_*u*_ when there are no measured data. As for the Typhoon Fung-Wong, the mean values of  *I*_*v*_ were equal to 0.89*I*_*u*_ based on the measured values. It showed that the measured results can well conform to this rule. In addition, there was an obvious correlation between the *I*_*u*_ and the *I*_*v*_.
2. The measured along-wind turbulence power spectrum density of Typhoon Fung-Wong coincided well with the Kaimal spectrum in general, especially in the middle frequency region from 0.04 Hz to 0.25 Hz. However, the measured vertical turbulence power spectrum density of Typhoon Fung-Wong did not fit well with the Panofsky spectrum.
3. In general, the acceleration RMS values of both the deck and the stayed cables become larger as the wind speed increases. The measured data also exhibit strong randomness. Therefore, in order to obtain the relationship between RMS values and wind speed, it is essential to preprocess the data. For instance, decompose the wind speed vectors.
4. The calculated dynamic characteristics are in good agreement with the measured ones, validating the reliability of the FE model. It provides baseline FE model for the following comparisons between the calculated and the measured results. The relatively large difference in lateral bending frequency indicates that the precise simulation of wind-resistant supports is important for the modal analysis of long-span bridges.
5. During the Typhoon Fung-Wong, all of the natural frequencies of the first mode are lower than those obtained during the normal windy condition, while all of the natural damping ratios of the first mode during Typhoon Fung-Wong are higher. In addition, the identified *ζ* values corresponding to the torsional vibration modes are relatively large, which should be paid special attention to when conducting dynamic analysis on SCB.
6. The measured vibration characteristics of upstream and downstream cables demonstrate remarkable regularity, but there was a great discrepancy on the contributions of each mode frequency component. It implies that the wind environments of the windward and leeward side are different. After the wind flows past the windward cables, the wakes affect the leeward wind environment.
7. The comparison between the measured data and the results from other studies reveals the effectiveness of dampers installed around anchor ends of the cables. Therefore, dampers can be used to reduce the vibration acceleration of cables during the strong winds, especially the in-plane acceleration components.

## Figures and Tables

**Table 1:** Natural frequencies and modal damping ratios of the SCB.

Vibration modes	1st sym L	1st sym V	1st sym T	1st anti-sym T
*f*_mf_ (Hz)	0.112	0.186	0.542	0.611
*f*_mn_ (Hz)	0.118	0.195	0.558	0.629
*f*_*c*_ (Hz)	0.107	0.185	0.544	0.603

*ζ*_*f*_ (%)	3.65	3.24	1.23	1.26
*ζ*_*n*_ (%)	2.73	2.41	0.80	0.85

*E*_*f*1_ (%)	−5.4	−4.8	−3.0	−2.9
*E*_*f*2_ (%)	4.5	0.5	−0.4	1.3
*E*_*ζ*_	25.2	25.6	34.9	32.5

Note: sym: symmetric; L: lateral; V: vertical; T: torsional; *f*_mf_: frequency measured during Typhoon Fung-Wong; *f*_mn_: frequency measured during normal wind; *f*_*c*_: calculated frequency based on FE method; *ζ*_*f*_: damping ratio identified during Typhoon Fung-Wong; *ζ*_*n*_: damping ratio identified during normal wind; *E*_*f*1  _= [(*f*_mf_ − *f*_mn_)/*f*_mf_] × 100%; *E*_*f*2_ = [(*f*_mf_− *f*_*c*_)/*f*_mf_] × 100%; *E*_*ζ*_ = [(*ζ*_*f*_ – *ζ*_*n*_)/*ζ*_*f*_] × 100%.
